# Discrimination of DNA Methylation Signal from Background Variation for Clinical Diagnostics

**DOI:** 10.3390/ijms20215343

**Published:** 2019-10-27

**Authors:** Robersy Sanchez, Xiaodong Yang, Thomas Maher, Sally A. Mackenzie

**Affiliations:** Departments of Biology and Plant Science, The Pennsylvania State University, University Park, PA 16802, USA; xzy50@psu.edu (X.Y.); twm118@psu.edu (T.M.)

**Keywords:** DNA methylation, signal detection, machine learning, leukemia, autism, clinical diagnostic

## Abstract

Advances in the study of human DNA methylation variation offer a new avenue for the translation of epigenetic research results to clinical applications. Although current approaches to methylome analysis have been helpful in revealing an epigenetic influence in major human diseases, this type of analysis has proven inadequate for the translation of these advances to clinical diagnostics. As in any clinical test, the use of a methylation signal for diagnostic purposes requires the estimation of an optimal cutoff value for the signal, which is necessary to discriminate a signal induced by a disease state from natural background variation. To address this issue, we propose the application of a fundamental signal detection theory and machine learning approaches. Simulation studies and tests of two available methylome datasets from autism and leukemia patients demonstrate the feasibility of this approach in clinical diagnostics, providing high discriminatory power for the methylation signal induced by disease, as well as high classification performance. Specifically, the analysis of whole biomarker genomic regions could suffice for a diagnostic, markedly decreasing its cost.

## 1. Introduction

Cytosine DNA methylation (5-methylcytosine; 5mC, CDM) is one of the most well-studied epigenomic marks and mechanistically understood epigenetic modifications to date [1,2]. It plays important roles in various biological processes, including X-chromosome inactivation, genomic imprinting, transposon suppression and transcriptional regulation [3]. Technological improvements and dramatic cost reductions for whole-genome sodium bisulfite sequencing (WGBS) of DNA have opened the door to the quantitative measurement of DNA methylation at a single base resolution, with datasets now available from numerous species.

In humans, significant methylation differences are observed in the white blood cells of matched monozygotic and dizygotic twins [4], and significant intra- and inter-population differential methylation has been identified in a study of three human populations (Caucasian-American, African-American and Chinese-American) [5]. Likewise, DNA methylation levels vary considerably in samples experiencing changes during development [6,7] or in response to environmental change [5,8].

Translation of the advances in DNA methylation to clinical and personalized medical contexts has been proposed [9,10,11]. Evidence of epigenetic alterations induced by disease emphasizes the significant potential value of high-resolution methylation pattern analysis. However, proper translation of this knowledge for diagnostics depends upon the development of genome-wide techniques for the rapid and robust identification of specific epigenetic alterations associated with the disease [9,12].

Currently, there are numerous available bioinformatics tools to estimate the methylation status of nearly every cytosine position within a whole-genome bisulfite sequencing dataset. These tools are generally supported by an application of robust statistical approaches; DSS [13], BiSeq [14], and methylKit [15], among others, are ones that apply generalized linear regression (beta-regression) in the estimation of differentially methylated positions (DMPs). In addition, methylKit provides the option to apply Fisher’s exact test. Alternatively, methylpy [16] bases its estimation on the implementation of the root-mean-square test (RMST) [17]. Most of these approaches do not incorporate the influence of natural stochasticity (randomness) to their models, limiting their resolution predominantly to genomic regions with the highest probability of having undergone a methylation change [1,18,19,20]. As a consequence, these approaches do not have the ability to distinguish the DMP signal associated with a specific treatment (or disease) from DMPs deriving from natural variation within control (or heathy) individuals, and are thus not suitable for clinical diagnostics. We suggest that methylation variation in diagnostics is essentially a signal detection problem, equivalent to a binary classification problem for discriminating healthy versus diseased individuals. Signal detection theory provides the methodological framework to address this type of detection problem, evidenced in its common application to clinical diagnostic tests, machine-learning (ML) approaches, and human communication technologies [21,22,23,24].

The probability of extreme methylation changes occurring spontaneously in a control group of samples by the stochastic fluctuations inherent to biochemical processes [25,26,27] and DNA maintenance [18], requires the discrimination of this background variation from a biological treatment signal. Regardless of environmental constancy, statistically significant methylation changes are found in control populations with probability greater than zero [25,28,29]. These system fluctuations, inherent in biological stochastic processes [30,31,32], comprise natural background variation detected in human methylomes [5,33]. By simulation, it is feasible to demonstrate that DMPs spontaneously arise in control populations, and that regulatory methylation signals also occur naturally in the control group [33].

Stochastic fluctuation of the methylation process is expected, since methylation regulatory machinery participates, not only in organismal adaptation to micro- and macro-environmental fluctuations [26,32], but also in transitions across different stages of organismal ontogenetic development. This variation must be factored into the construction of a methylation pipeline to be used in clinical diagnostics.

The need for the application of signal detection-based approaches in diagnostics was pointed out decades ago [34], and is standard practice in current implementations of clinical diagnostic tests [21,35,36]. Detection approaches are, by default, included in machine/statistical learning implementations for classification tasks [37], since the evaluation of classifier performance is basically a detection problem. Thus, the determination of the optimal cutoff (threshold) value at which signal can be discriminated from noise at an acceptable signal-to-noise ratio (i.e., maximum accuracy and sensitivity, lowest false discovery rate (FDR)) is equivalent to a classification problem, a direct corollary to diagnostic detection.

The natural spontaneous variation of DNA methylation in human populations [33] complicates the discrimination of the methylation signal from background variation. We address this issue by determining whether a given DMP detected in the treatment/patient population occurs within the control population as well, and the probability of observing it. Therefore, the focus is not on the identification of DMPs, but on whether these statistically significant changes occur with high probability (under the fixed experimental conditions) in only the treatment group. Here we address this problem in the context of signal detection theory and ML frameworks [21,22].

To illustrate the feasibility of our proposed approach in clinical diagnostics, results from simulation studies and tests of two available methylome datasets from leukemia [38] and autism [39] patients are presented and discussed. This approach is implemented in the R package Methyl-IT, available at GitHub: https://github.com/genomaths/MethylIT. The R scripts to reproduce these analyses are available at the PSU GitLab: https://git.psu.edu/genomath/MethylIT_examples.

## 2. Results

### 2.1. Detection of the Methylation Signal

**Figure 1:**
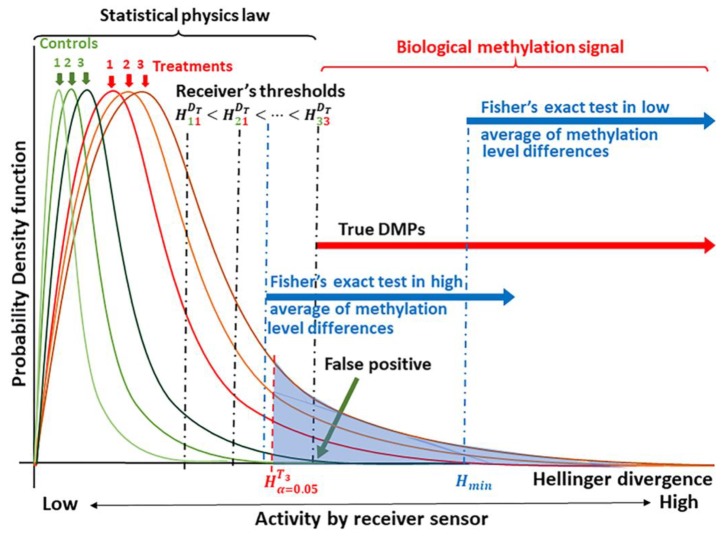
Schematic of the theoretical principle underlying signal detection applied to methylation analysis. Knowledge of the probability distribution for the methylation signal, here given as the Hellinger Divergence (*H*) of methylation levels, permits the identification of “true” differentially methylated positions (DMPs) [21,22]. Critical values are used to discriminate between a biological methylation signal and the molecular thermal noise generated by biochemical processes and conforming to laws of statistical physics [25,26,27,40]. Next, signal detection is designed to identify an optimal cutoff value, (here denoted as $H_{33}^{D_{T}}$) to discriminate the methylation signal induced by the treatment from the natural background variation [5,33]. Empirical comparisons allow the placement of Fisher’s exact test for the discrimination of DMPs.

A basic requirement for the application of signal detection is knowledge of the probability distribution for background noise (noise plus signal) [21]. Models for the probability distributions of information divergences for methylation levels where derived on the basis of statistical physics [40]. The basic concept for the application of signal detection theory is shown in Figure 1.

Biologically meaningful methylation changes are distinguishable in the tails of the probability distributions for methylation signal, which are experimentally derived from each individual. In the current case, the signal is given in terms of the Hellinger Divergence (*H*) of methylation levels (Figure 1). For a given level of significance α (Type I error probability, e.g., α = 0.05), cytosine positions with $H>H_{\alpha =0.05}$ can be selected as sites carrying a potential biological signal (shown as the blue shaded region under the curve in Figure 1). The true signal is detected based upon an optimal cutoff value, which can be estimated from a receiver operating characteristic (ROC) using different optimized indices or using different ML approaches [23,24,36,41]. The probability that a DMP is not induced by the treatment is designated as the probability of a false alarm (*P_FA_*, false positive, Figure 1).

As suggested in Figure 1, we postulate that most of the observed methylation changes are induced by stochastic fluctuations that serve to stabilize the DNA molecule, and consequently, can be explained within a statistical physics context. Statistical physics, however, cannot explain Hellinger Divergence values above the cut point $H_{33}^{D_{T}}$. These would comprise methylation changes induced by the methylation regulatory machinery.

The stochasticity of methylation regulatory machinery effects is presumed to reflect system heterogeneity; cells from the same tissue are not necessarily in the same state, and therefore, corresponding cytosine sites differ in their methylation status. Consequently, overall organismal response is conveyed as a statistical outcome that distinguishes the regulatory methylation signal from statistical background “noise”.

Estimation of optimal cutoff value for the signal is an additional step to remove any remaining potential methylation background noise with probability 0≤α≤0.05. We define as a methylation signal (DMP) each cytosine site with Hellinger Divergence values above the cut point (shown in Figure 1 as $H_{33}^{D_{T}}$). Each DMP-associated signal may or may not be represented within a DMP derived by Fisher’s exact test (or other current tests).

The statistical tests generally applied to methylome analysis were not designed to discriminate the methylation signal from background variation, so that their sensitivity is lower than that provided by a signal detection approach. As illustrated in Figure 1, at a low average of methylation level differences, cytosine positions with *H* values between $H_{33}^{D_{T}}$ and $H_{min}$ carry a methylation signal, but a Fisher’s exact test can only detect signals in cytosine positions with *H* values greater than $H_{min}$. The difference in resolution is illustrated. An analogous situation can be observed for any other statistical test that does not include information on the signal probability distributions from the control and treatment groups.

**Figure 2:**
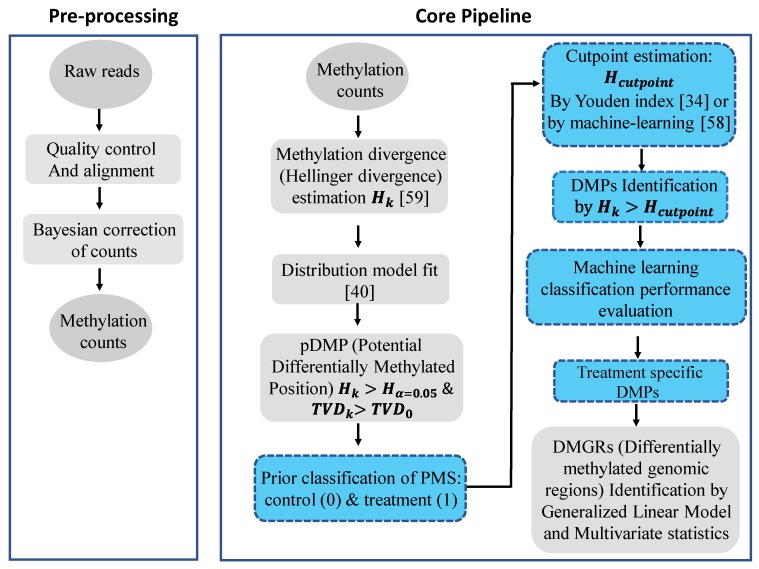
Flow diagram of the signal detection and machine learning approach applied in our methylation analysis study. The approach is implemented in the R package Methyl-IT, which is available at GitHub: https://github.com/genomaths/MethylIT. Ovals represent input and output data, squares represent processing steps, with signal detection processing and machine learning steps highlighted in blue. For the machine learning classification performance evaluation, data from independent individuals in the same population are preferred. The final step shown (DMGRs) is not included in the current study, but is an analytical step that can be accomplished with Methyl-IT. Related references are cited accordingly for each key step.

A flow chart of the signal detection and machine learning approach applied to implement the model discussed above is given in Figure 2. Briefly, raw bisulfite whole genome DNA sequencing reads are aligned by Bismark [42], methylation count (COV) files are extracted and read into R software, and Hellinger Divergence is calculated by using the pool of methylation counts from control (healthy) individuals as a reference. Potential differentially methylated positions (pDMPs) are estimated based upon critical values of *HD*_α=0.05_ and *TVD* cut off points estimated for each individual from the best fitted probability distribution model. Final DMPs are derived from the set of pDMPs by estimating the optimal cut-off threshold for *HD* based on the Youden index or ML classification performance.

The performance of the model classifier is evaluated by cross-validation in Methyl-IT. A Monte Carlo cross-validation helps to prevent an overfitting issue, providing a more accurate indication of how well the model generalizes to unseen data [43]. Model overfitting occurs when a model learns the features and noise in the training data to the extent that it negatively impacts performance of the model on new data. This means that the noise or stochastic fluctuations in the training data are learned as features by the model [44]. In Methyl-IT, an extensive Monte Carlo resampling (default 999) splits the dataset into training (default 60%) and testing sets reiteratively. Each time, several classification performance indicators are computed: Accuracy, kappa statistic, sensitivity, specificity, positive predictive and negative predictive values, detection rate and false discovery rate. The final report includes the means of all indicators and their bootstrap confidence intervals. In addition, a prediction function is provided to evaluate the model classifier performance on an external dataset.

### 2.2. Simulation Study

A summary of the datasets generated for simulation experiments is presented in Table 1. The analyses involved the application of the built-in Monte Carlo cross-validation of the ML binary classification model and the validation on external data. Two main sets of simulation experiments were accomplished. For experiments 1 to 3 (small sample size), ML classification models were built on a training set, and Monte Carlo cross-validation was performed for each of them. External validations for the ML model obtained in the training set from 1 to 3 were performed on datasets 4 to 6 (larger sample size, Table 1). Similarly, ML classification models were built on a training set from datasets 7 to 9 (sample size 50) and validation was accomplished as described previously. All control and treatment DMPs from each subset, training or test subset (from all samples) were used for model building and cross-validation, and likewise for the external validation, since the predictions were performed not for samples, but for cytosine sites.

Classification performances shown in Table 1 suggest that if read counts come from the same populations, (control/healthy or treatment/patient), that were used to build the binary ML classification model, then the model is able to properly distinguish, with high accuracy, control DMPs from treatment. Moreover, the robustness of the signal-detection-ML approach is retained even when the model is built from a small sample size. Thus, the signal-detection-ML approach relies not on sample size, but on the genome-wide probability distribution of the Hellinger Divergence.

The population homogeneity assumed in the simulation studies summarized in Table 1 is not always the case for actual human data, where patients can belong to different subpopulations. In the next section we show that in such a situation the signal-detection-ML remains sufficiently robust.

As suggested in Figure 1, the signal detection-ML proposed here confronts two binary classification problems to solve at once: (1) The classification of cytosine sites into two classes, DMPs and non-DMPs, and; (2) the classification of DMPs into two classes, control and treatment DMPs. Results suggest that the false positive rate (*FPR*) on the simulated (external) datasets for the classification problem (1) remains below 0.05 (Supplementary Table S1).

**Figure 3:**
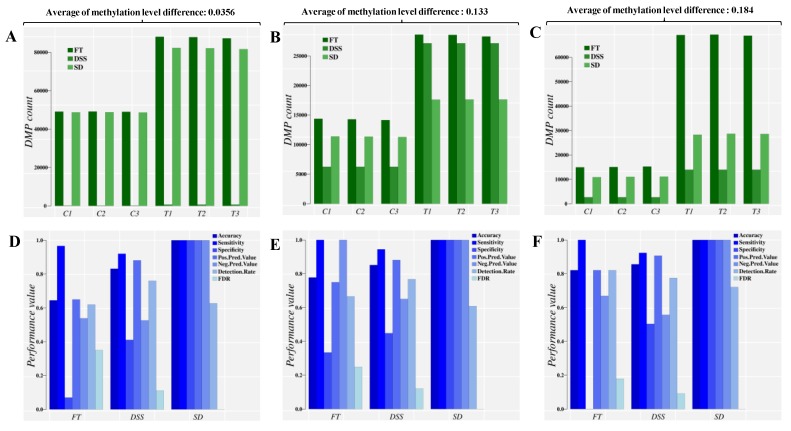
Direct comparison of the classification performance for control and treatment DMPs based on three different approaches and at three different averages of absolute methylation level differences (mild difference, 0.0356, medium difference, 0.133 and large difference, 0.184). Panels (**A**–**C**) report the sum of DMPs detected, while panels (**D**–**F**) report the sum of main classifier performance indicators. An extended list of performance indicators is given in the supplementary information. C1-C3: control individuals. T1–T3: Treatment individuals. FT: Fisher’s exact test, used by methylKit [15]. DSS: An R package that uses generalized linear regression and Wald Test in the DMP identification [13]. SD: Signal detection approach, implemented in Methyl-IT.

Results from the simulation study involving different approaches to estimate DMPs are shown in Figure 3. Three approaches were considered: (i) Fisher’s exact test (FT), (ii) differential methylation analysis with the DSS R package [45], and (iii) signal detection (SD) implemented in the R package Methyl-IT. The analyses considered three averages of (absolute) methylation level difference: 0.356, 0.133 and 0.184. Details are given in Methods.

The three approaches were able to identify DMPs in the control group as predicted. Such detection is implicit (by construction) in the SD approach. An optimal cut off estimation to distinguish the control DMPs from treatment was accomplished for each approach as well, classifying the set of DMPs into control and treatment groups.

Results indicate that the application of a statistical test alone, ignoring the distribution of methylation signals, leads to an overestimation of DMPs in both populations, control and treatment (Figure 3A–C, FT bars). For any population where the average methylation level differences are relatively high, the risk of DMP overestimation trends higher than in a population with low average methylation (dark-green bars, FT, Figure 3A–C). This overestimation can be eliminated by introducing information from signal detection (Figure S1A–C, FT.SD bars). Note that all DMPs detected by the Fisher test and DSS were valid by statistical terms. The 95% empirical quantile ($TV_{95}$) of absolute difference in methylation levels (total variation distance, *TV*) was applied as the cutoff value in each approach, so that only sites with $TV\geq TV_{95}$ were included in the analyses (minimum value $TV_{95}=0.35$ in the case of 0.0356 average methylation level difference). This approach gives a range of values detectable by any statistical test designed to test counts. For the signal detection approach (SD bar in Figure 3A–C), the number of cytosine sites with ($TV\geq TV_{95}$) that are identified as DMPs depends on the optimal cutoff value estimated based on signal detection criteria [21,22,34,35,36,37], i.e., the information divergence value that leads to best DMP classification performance.

For the proper discrimination of DMPs into control and treatment groups, machine learning classifiers were used (as well as the Youden Index [34], see Methods). The optimal cutoff value estimated based on signal detection criteria was derived for each approach, which in the case of SD is a default step, as indicated above. The best classification performance obtained for each simulation approach is shown in Figure 3D–F. The application of a statistical test alone leads to low classification performance, based on frequency of reporting control DMPs as treatment-induced. Since the optima HD cut-off value increases as the genome-wide average of methylation level differences increases, the number of control DMPs also found in the treatment dataset increases as well. This outcome does not reflect a failure of the Fisher Test or DSS statistical approaches, since it is a typical signal detection and ML (classification) problem.

### 2.3. Analyses of Experimental Datasets

Signal detection was further evaluated with two experimental DNA methylation datasets: (i) Chromosome 9 from patients with pediatric acute lymphoblastic leukemia (PALL, including CD19 control cells [38]) and (ii) placental tissue of typically developing and autistic children [39].

#### 2.3.1. Analysis of PALL Dataset

**Figure 4:**
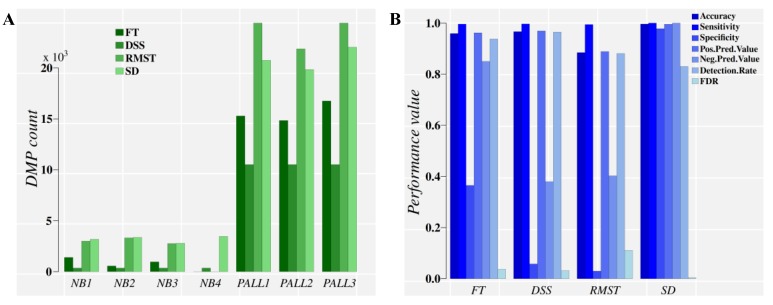
Direct comparison of classification performance for the control and treatment DMPs obtained on Chromosome 9 from the pediatric acute lymphoblastic leukemia (PALL) dataset by four methylation analysis approaches. Panel (**A**), DMP counts. Panel (**B**), classification performance evaluation. For a diseased individual, a diagnostic is based on the high classification performance obtained for hundreds of thousands of DMPs (when the whole methylome is analyzed) that are discriminated from methylation background variation found in the control population. NB1–NB4: Healthy individuals. PALL1-3: PALL patient individuals. FT: Fisher’s exact test, used by methylKit [15]. DSS: An R package that uses generalized linear regression and the Wald Test in the DMP identification [13]. RMST: Root-mean-square test, used by methylpy [16]. SD: Signal detection approach, implemented in MethylIT.

DMPs were estimated for control (four normal CD19+ blood cell donors) and patient (ALL cells from three patients) groups relative to a reference group of four independent normal CD19+ blood cell donors. Results of the methylation analysis and DMP classification performance are presented in Figure 4.

Figure 4A suggests that FT performs poorly in the PALL methylome dataset, partly due to the *p*-value adjustment required for multiple comparisons. This detail is evident from the FT.SD approach (Figure S2), which relies on the direct FT results with knowledge derived from signal detection (for the optimal cut point), and does not require *p*-value adjustment. RMST, a test with higher sensitivity than FT, was applied as well [16]. Again, resolution loss with RMST, by ignoring the signal probability distribution, comes in a lower classification performance than with approaches that include this information (SD in Figure 4B). The classification performance of DMPs derived from FT and RMST approaches are notably improved when the ML classifiers are fed information on the optimal cutoff divergence value and probability distribution of the methylation signals (FT.SD, RMST.SD, and SD, in Figure S2B).

#### 2.3.2. Analysis of Placenta from Typically Developing and Autistic Children

Twenty placenta methylomes from typically developing (TDP) and autistic (ADP) children were analyzed with the signal detection approach implemented in the Methyl-IT R package (https://github.com/genomaths/MethylIT). The methylome datasets were split into two groups. The first group (G1) with five TDP and four ADP samples, and a second (G2) with three TDP and nine ADP samples (details in Methods). Results are summarized in Table 2.

Placental DMPs from children with autism were distinguishable from the control (TDP). Moreover, model classifiers from each group were able to predict with high accuracy DMPs from the other group. In this case, the best performance was obtained for predicting DMPs from group G2 by using a model classifier built with a training set from group G1 (Table 1, G1 pred. G2), which likely indicates that, for a model classifier, group G1 has more balanced learning samples (TDP and ADP individuals) than G2.

#### 2.3.3. Methylation Signal Association with Genes Involved in Disease Development

**Figure 5:**
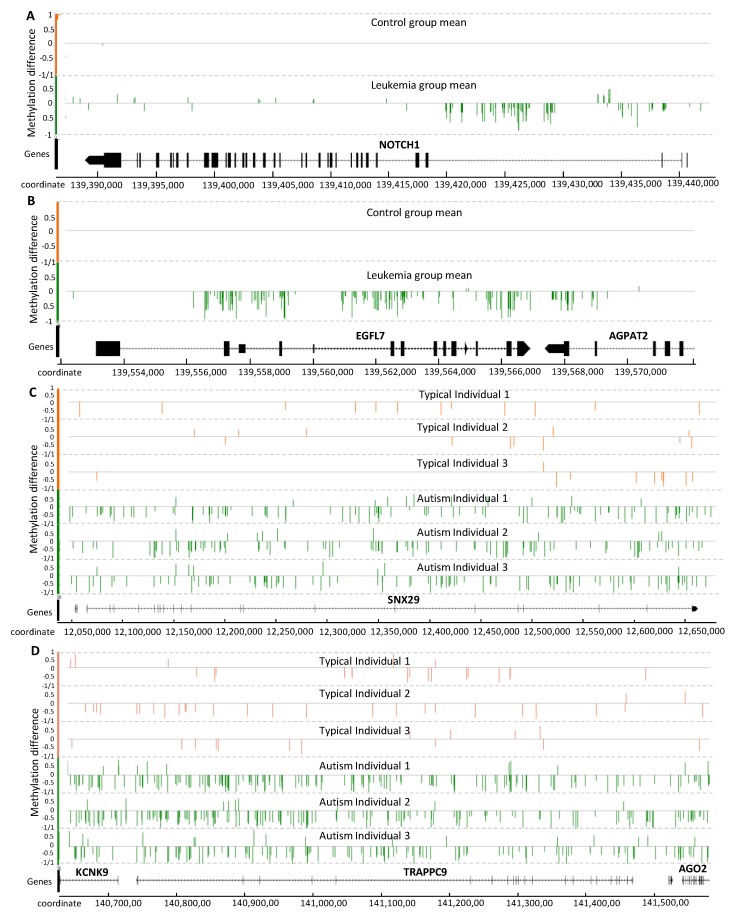
Distribution of the methylation signal on gene-body for seven relevant genes reported to be associated with PALL (**A**,**B**) and autism (**C**,**D**). Methylation level difference at each cytosine is computed by (*mC*/(*mC* + *uC*))_each individual_ − (*mC*/(*mC* +*uC*)_average of all reference individuals_, with *mC* and *uC* denoting the numbers of methylated and unmethylated reads, respectively. Each cytosine is represented by one single vertical line. The Integrated Genome Browser (version 9.0.2) was used to generate this figure.

Results indicated that signal detection provided a high classification performance of DMPs. This outcome led us to assess DMP association with known genes involved in disease development. Although it is not the purpose of the current work to present an exhaustive analysis of genes targeted by a differential methylation signal, relevant examples are presented in Figure 5 to illustrate the distribution of this signal on genes.

Three relevant genes from chromosome 9, known to be involved in leukemia development, are shown in Figure 5A,B: *NOTCH1*, *EGFL7* and *AGPAT2*. *NOTCH1* is reported to be epigenetically regulated and proposed as a drug target for the treatment of T-cell acute lymphoblastic leukemia [46,47]. Interestingly, most of the detected methylation signal is concentrated within introns at the 3’ end (Figure 5A). *EPIDERMAL GROWTH FACTOR-LIKE DOMAIN 7 (EGFL7*) is also associated with cancer development [48], and the methylation signal detected in *EGFL7* covers most of the gene-body (Figure 5B). The *EGFL7* gene is reported to be a key factor for the regulation of the *EGFR* signaling pathway [49]. *EGFL7* is also a secreted angiogenic factor that can result in pathologic angiogenesis and enhance tumor migration and invasion via the *NOTCH* signaling pathway [50], a conserved intercellular signaling pathway that regulates interactions between physically adjacent cells.

*ACYLGLYCEROL-3-PHOSPHATE ACYLTRANSFERASE 2* (*AGPAT2*) promotes the survival and etoposide resistance of cancer cells under hypoxia [51]. The hypomethylation pattern observed in the gene-body region of *EGFL7* spans a substantial part of *AGPAT2* (Figure 5B).

Although the methylation signal induced by cancer has typically been reported as genome-wide hypermethylation, and this is the case for the PALL methylome as well, Figure 5A,B indicate that some relevant genes can experience hypomethylation.

The methylation signal within three genes reportedly associated with autism disorder are shown in Figure 5C,D: *SNX29*, *TRAPPC9*, and *KCNK9*. *SORTING NEXIN 29* (*SNX29,* also known as *RUN Domain-Containing Protein 2A, RUNDC2A*) is a gene previously associated with schizophrenia [52]. This locus has been reported as a differentially methylated locus and autism-associated gene in the US patent US20180142298A1. The gene *TRAFFICKING PROTEIN PARTICLE COMPLEX 9* (*TRAPPC9*) appears to function in neuronal cell differentiation and is reported as an autism susceptibility gene in the SFARI database (https://gene.sfari.org/). Mutations in this gene have been associated with autosomal-recessive cognitive disability, causing non-syndromic intellectual disability and speech disorder [53].

The *TRAPPC9* locus was identified by an extended hypomethylation signal region that spans two other genes of interest, *KCNK9* and *AGO2*, down- and up-stream, respectively (located on the negative strand). *POTASSIUM TWO PORE DOMAIN CHANNEL SUBFAMILY K MEMBER 9 (KCNK9)* encodes a typical potassium channel protein that is especially abundant in brain neurons. *KCNK9* is a maternally-expressed and imprinted gene, so that only the maternal gene copy is active [54]. Mutations in this gene produce *KCNK9 imprinting syndrome* [54]. *KCNK9* has been reported within a list of gene expression biomarkers for autism in patent US20130210650A1.

Finally, *ARGONAUTE RISC CATALYTIC COMPONENT 2* (*AGO2*) is a gene required for RNA-mediated gene silencing (RNAi) by the RNA-induced silencing complex (RISC). AGO2 plays a key role in neuronal plasticity [55]. RISC proteins *Dicer* and *Ago2* localize to distal neuronal compartments, indicating a spatial, functional role for microRNAs [56]. In the current set of placental methylomes from autistic children, the three genes *KCNK9, TRAPPC9* and *AGO2*, are located in a contiguous hypomethylated region. This result contrasts with a recent report addressing uncertainty about the diagnostic value of AGO2 gene expression in blood samples from autistic patients, where the authors concluded that further studies are required [57].

## 3. Discussion

In this work we emphasize the need for the application of detection theory and machine learning on the discrimination of the DNA methylation signal from background population variation for clinical diagnostic purposes. As a consequence of natural background variation, DMPs are detected not only in the patient population, but also in any set of control individuals [33]. As a result, the diagnosis problem is essentially a classification problem.

The methylation signal is often altered in patients suffering from disease, and Methyl-IT can be effective for the diagnosis of patients based upon signal detection. As highlighted in earlier reports [21] and [22], signal detection theory provides the methodological framework to effectively confront a detection problem.

Hence, regardless of the statistical test applied to identify this methylation signal, the application of detection theory and machine learning is valid to discriminate endogenous background signal (DMPs) from that induced by the treatment or disease-state in patients. A proper diagnostic requires evaluation with suitable classification performance measurements: High accuracy, sensitivity and specificity values, low FDR and other performance indicators commonly reported (Table 1 and Table 2, Figure 3 and Figure 4).

Proper application of signal detection requires knowledge of the probability distribution of the background noise in a system [21,22]. The probability distribution of the signal can be inferred from the experimental datasets, control and treatment [40]. This information provides a strong predictive value, and one can infer the probability of signal values in the control and treatment (patient population) that are not observed in the available datasets. Consequently, this signal probability distribution allows an estimation of the optimal cutoff value to discriminate signal induced by treatment or disease state from background.

Current methylation analysis methods that employ FT, RMST and DSS are limited to direct multiple comparisons of control versus treatment to search for significant statistical differences at each cytosine site in methylome datasets. This approach does not allow for predictive modeling, since the statistical tests are only designed to evaluate differences, not to serve as model classifiers. Moreover, these statistical tests do not directly evaluate background variation.

Proper measurement of the methylation signal requires a reference sample from which an information divergence of methylation levels can be measured for control and treatment samples. In this way, signals derived from background variation and that induced by the treatment are measured with the same origin of coordinates.

Simulation studies showed that, depending on the statistical approach (FT or DSS) and *TV* average, ignoring natural background variation can lead to a misestimation of the methylation signal (Figure 2). In all scenarios, DMPs detected by FT and DSS approaches were valid in statistical terms. However, the signal-to-noise issue comprises a post-DMP detection problem.

As shown in Figures S1 and S2, the classification performance obtained for the FT and RMST approaches notably improve after being fed the ML classifier with information derived from the methylation signal probability distribution and the detection step (the optimal cutoff *HD* value). Therefore, invoking the parsimony principle, we assume that signal detection and machine learning classifiers are sufficient [58].

The combination of signal detection and machine learning appears to be adequately robust to perform diagnostics on experimental/clinical datasets displaying either a low or high average of absolute methylation level differences (*TV*, Figure 3). To test empirical examples of these natural scenarios, two patient datasets were considered, pediatric acute lymphoblastic leukemia (PALL) and placental tissue from autistic children. Both datasets displayed a relatively high natural background of *TV* average in the control population, and a weaker methylation signal in placental tissue from autistic children than in the PALL patient dataset. Results were consistent with those obtained in the simulation study (Figure 4 and Table 1). The PALL dataset demonstrated that regardless of any statistical test applied, signal detection was required to reach the high classification performance required for clinical diagnostics (Figure 4 and Figure S2). Pronounced signal differentiating control and disease state was observed in association with loci known to be altered during cancer development.

Encouraging results were also obtained with placental tissue from autistic children (Table 2). This dataset was selected to reflect common sources of variation inherent to clinical studies, including diagnoses from different doctors, tissue samples reflecting collection feasibility rather than site of abnormality, and modest bisulfite sequencing depth per patient sample. In spite of the high natural background variation detected in placental samples, model classifiers which are built in training sets of one group of patients, independently analyzed with respect to control samples, could be applied to predict the entire set of individual DMPs (control and patient) from the other group (cases “G2 pred. G1” and “G1 pred. G2”).

It is important to emphasize the value of the classification performance evaluation, which is built into the Methyl-IT package as a validation procedure. It would not be advisable for users to continue an analysis if the classification performance is poor, even when optimal parameters are used. In the case presented, the robustness of the classification model built on Group G1, previously evaluated by cross-validation, was corroborated by the high classification performance reached on predicting the whole G2 (external data). Admittedly, further studies are needed to properly establish and validate a clinical diagnostic test for autism based on methylome data from placental tissue, but results suggest a potential avenue to address this seemingly intractable challenge.

Epigenetic variation can influence biologically relevant networks that are specific to each cell type, often occurring near genes that have functional relevance to the cell type [33]. As shown in Figure 5, we were able to identify relevant genes displaying differential methylation signals distinguishable from the natural background variation and putatively associated with disease, several proposed as drug targets for patient treatment or reported as biomarker candidates.

These observations are not sufficient alone to conclude a direct disease relationship, but the reproducibility of these data, combined with machine learning-based validation, provide a compelling argument for their further study.

## 4. Materials and Methods

### 4.1. Divergences of Methylation Levels

Information divergences of methylation levels, total variation distance $\hat{T}V_{d}\left({\hat{p}}_{c},{\hat{p}}_{t}\right)$ and Hellinger Divergence $\hat{H}\left({\hat{p}}_{c},{\hat{p}}_{t}\right)$, were estimated for control and treatment (disease stage) relative to a reference virtual individual. The reference sample was built from a subset of individuals from the control population that were not included as our control.

In a Bayesian framework assuming uniform priors, the methylation level ${\hat{p}}_{i}$ can be defined as: ${\hat{p}}_{i}=\left(n_{i}^{mC}+1\right)/\left(n_{i}^{mC}+n_{i}^{C}+2\right)$, where $n_{i}^{mC}$ and $n_{i}^{C}$ represent the numbers of methylated and non-methylated read counts observed at the genomic coordinate i, respectively. We estimate the shape parameters α and β from the beta distribution minimizing the difference between the empirical and theoretical cumulative distribution functions (ECDF and CDF, respectively):

(1) $$P\left(p|\alpha ,\beta \right)=\frac{p^{\alpha -1}{\left(1-p\right)}^{\beta -1}}{B\left(\alpha ,\beta \right)}$$

where B(α,β) is the beta function with shape parameters α and β. Since the beta distribution is a prior conjugate of binomial distribution, we consider the parameter *p* (methylation level) in the binomial distribution as randomly drawn from a beta distribution. The hyper-parameters α and β are interpreted as pseudo counts. Then the mean $E\left[p_{i}|D\right]={\hat{p}}_{i}$ of the methylation levels $p_{i}$, given the data *D*, is expressed by:

(2) $${\hat{p}}_{i}=\frac{\alpha +n_{i}^{mC}}{\alpha +\beta +n_{i}^{mC}+n_{i}^{C}}$$

The methylation levels at the cytosine with genomic coordinate i are then estimated according to this equation.

As shown in Figure S4, total variation distance $TV_{d}\left(p_{c},p_{t}\right)$ sets the natural metric in the probabilistic space (p,1−p), and it is defined the absolute value of methylation level differences:

(3) $$\hat{T}V_{d}\left({\hat{p}}_{i}^{c},{\hat{p}}_{i}^{t}\right)=\left|{\hat{p}}_{i}^{t}-{\hat{p}}_{i}^{c}\right|$$

Notice that $\hat{T}V_{d}\left(p_{c},p_{t}\right)$ is the Manhattan distance in the space (p,1−p). Biostatisticians and biologists in general are familiar with the root-square transformation of the original variables: $\sqrt{x}$. The root-square transformation maps the space (p,1−p) into the new space $\left(\sqrt{p},\sqrt{1-p}\right)$. The Euclidean distance $d_{E}\left(\sqrt{p_{c}},\sqrt{p_{t}}\right)$ is a ‘natural’ metric to introduce into the space $\left(\sqrt{p},\sqrt{1-p}\right)$, which turns out to be the Hellinger Divergence of the original variables (Figure S4). The square of the Euclidean distance $d_{E}{\left(\sqrt{p_{c}},\sqrt{p_{t}}\right)}^{2}$ in the space $\left(\sqrt{p},\sqrt{1-p}\right)$ corresponds to the Hellinger Divergence $\hat{H}\left({\hat{p}}_{c}^{},{\hat{p}}_{t}^{}\right)={\left(\sqrt{{\hat{p}}_{t}^{}}-\sqrt{{\hat{p}}_{c}}\right)}^{2}+{\left(\sqrt{1-{\hat{p}}_{t}^{}}-\sqrt{1-{\hat{p}}_{c}^{}}\right)}^{2}$ in the space (p,1−p).

Here, however, the Hellinger Divergence will be used as given in reference [59], which is defined based on the estimated methylation levels ${\hat{p}}_{i}$ at given cytosine site *i* as:

(4) $$\hat{H}\left({\hat{p}}_{i}^{c},{\hat{p}}_{i}^{t}\right)=w{\left(\sqrt{{\hat{p}}_{i}^{c}}-\sqrt{{\hat{p}}_{i}^{t}}\right)}^{2}+{\left(\sqrt{1-{\hat{p}}_{i}^{c}}-\sqrt{1-{\hat{p}}_{i}^{t}}\right)}^{2}$$

where $w_{i}=2\frac{m_{i}^{c}m_{i}^{t}}{m_{i}^{c}+m_{i}^{t}}$, $m_{i}^{t}=n_{i}^{mC_{c}}+n_{i}^{C_{c}}+1$, and $m_{i}^{t}=n_{i}^{mC_{t}}+n_{i}^{C_{t}}+1$.

According with Equation (4), not only the methylation levels are considered in the estimation of *H*, but also the control and treatment coverage at each given cytosine site. Under the null hypothesis of non-difference between distributions ${\hat{p}}_{i}^{c}$ and ${\hat{p}}_{i}^{t}$, Equation (4) asymptotically has chi-square distribution with one degree of freedom, which sets the basis for a Hellinger chi-square test (HCT) [59].

Distance $\hat{T}V_{d}\left({\hat{p}}_{c},{\hat{p}}_{t}\right)$ and Hellinger Divergence (as given in Equation (4)) hold the inequality: $\hat{T}V_{d}\left({\hat{p}}_{i}^{c},{\hat{p}}_{i}^{t}\right)\leq \sqrt{2}{\hat{H}}_{d}\left({\hat{p}}_{i}^{c},{\hat{p}}_{i}^{t}\right)$, where ${\hat{H}}_{d}\left({\hat{p}}_{i}^{c},{\hat{p}}_{i}^{t}\right)=\sqrt{\hat{H}\left({\hat{p}}_{i}^{c},{\hat{p}}_{i}^{t}\right)/w_{i}}$ is the Hellinger Distance, a direct consequence of the Cauchy-Schwarz Inequality.

Only cytosine sites with methylation level differences ($\hat{T}V_{d}$) greater than a cut-off value were included in the analysis.

### 4.2. Non-Linear Fit of Distribution Functions

The cumulative distribution functions (CDF) for $H_{k}\left(p_{k}^{c},p_{k}^{t}\right)$ can be approached by a Weibull distribution model:

(5) $$P\left(H_{k}\leq H^{0}|\alpha ,\lambda ,\mu \right)=1-e^{{\left(\frac{H_{k}-\mu }{\lambda }\right)}^{\alpha }}$$

where α, β, and μ are the parameters shape, scaling, and location, respectively, or the gamma distribution:

(6) $$P\left(H_{k}\leq H^{0}|\alpha ,\beta ,\mu \right)=\frac{\gamma \left(\alpha ,\beta \left(H_{k}-\mu \right)\right)}{\Gamma \left(\alpha \right)}$$

where Γ(α) is the gamma function. $\gamma \left(\alpha ,\beta \left(H_{k}-\mu \right)\right)$ is the lower incomplete gamma function with shape parameters α and β, and location parameter μ. Model parameters are estimated by non-linear regression analysis of the ECDF ${\hat{F}}_{n}\left({\hat{H}}_{k}\leq H^{0}\right)$ versus ${\hat{H}}_{k}\left({\hat{p}}_{i}^{c},{\hat{p}}_{i}^{t}\right)$. The ECDF of the variable ${\hat{H}}_{k}$ is defined as:

(7) $${\hat{F}}_{n}\left({\hat{H}}_{k}\leq H^{0}\right)=\frac{\mathrm{number}\;\mathrm{of}\;\mathrm{CDMs}\;\mathrm{in}\;\mathrm{the}\;\mathrm{samples}\;\mathrm{with}\;{\hat{H}}_{k}\leq H^{0}\;}{n}=\frac{1}{n}\sum_{k=1}^{n}1_{{\hat{H}}_{k}\leq H^{0}}$$

where $1_{{\hat{H}}_{k}\leq H^{0}}=\{\begin{array}{c} \;1\;\mathrm{if}\;{\hat{H}}_{k}\leq H^{0}\;\\ 0\;\mathrm{if}\;{\hat{H}}_{k}>H^{0} \end{array}$ is the indicator function. Function ${\hat{F}}_{n}\left({\hat{H}}_{k}\leq H^{0}\right)$ is easily computed (for example, by using function “*ecdf*” of the statistical computing program R).

### 4.3. Detection of the Methylation Signal

As for any signal in nature or treatment induced, a suitable detection of the methylation signal is based on the knowledge of its probability distribution. The basic idea behind the application of signal detection is illustrated in Figure 1. Critical values $H_{\alpha =0.05}$ are estimated from the best fitted model (Equations (5) or (6)) for each individual sample from the control and treatment group. Depending on the average of methylation on the populations under screening, the true signal would be found at the right of the highest observed critical value $H_{\alpha =0.05}$. A cytosine position with a Hellinger Divergence value *H* greater than the critical value $H_{\alpha =0.05}$ is considered for further downstream analyses. A further step to estimate an optimal cutoff value of *H* is required. Although *H* is used here, other information divergences can be used as well.

For an estimation of an optimal cutoff value of *H,* three approaches were taken: (1) Based on the estimation of the Youden Index [34], (2) Based on the posterior classification probabilities of the potential signal (potential DMPs) into two classes (given by a model classifier), from control and from treatment, and (3) Based on the posterior classification probabilities derived from a gamma mixture model. Next, cytosine positions with *H* values greater than the cutoff value are considered DMPs, regardless of which group they belong to, control or treatment.

For the analysis with the DSS R package, methylation count (COV) files were read into R and prepared by the *makeBSseqData* function (DSS), DMPs then were computed using *DMLtest* function (DSS) without smoothing at *p*-value < 0.05.

### 4.4. DMP Prediction Based on Machine Learning Model Classifiers

The following model classifiers were tested for DMP predictions: PCA+LDA, PCA + QDA, PCA + logistic, and logistic models. That is, a principal component analysis (PCA) is applied on the original raw matrix of the data and then the derived principal components are used in a further linear/quadratic discriminant analysis (LDA/QDA). A scaling step is applied to the raw matrix of this data before the application of the mentioned procedure, which is not applied for the logistic model. Here, PCA will yield new orthogonal (non-correlated) variables, the principal components, which prevent any potential bias effect originated by correlation or association of the original variables.

Four predictor variables were considered: $TV_{d}$, *H*, relative position of the cytosine site in the chromosome, and the logarithm base two of the probability to observe a Hellinger Divergence value *H* greater than the critical value $H_{\alpha =0.05}$: $log_{2}P\left(H>H_{\alpha =0.05}\right)$.

All data analysis was performed with the R package MethylIT version 0.3.2 available at GitHub (https://github.com/genomaths/MethylIT), where several user guide examples illustrate the application of MethylIT downstream methylation analysis.

### 4.5. Simulations

Twelve simulated datasets of methylated cytosines were generated based on three different averages of absolute methylation level differences: mild difference, 0.0356, medium difference, 0.133 and large difference, 0.184, and with different samples size (Table 1). Simulated data were generated using the function *simulateCounts* from the R package *MethylIT.utils* (https://github.com/genomaths/MethylIT.utils). Methylation coverages (minimum 10) were generated from a negative binomial distribution with the function *rnegbin* from the R package *MASS*. This function uses the representation of the negative binomial distribution as a continuous mixture of Poisson distributions with Gamma distributed means. Prior methylation levels are randomly generated with beta distribution using the *Beta* function from R package “*stats*”, and posterior methylation levels are generated according to Bayes’ Theorem.

The fact that each dataset of read counts was sampled from the same populations, control or treatment, does not mean that the individual samples will have the same probability distribution for the Hellinger Divergences of the methylation levels. Simulation was performed under the standard clinical assumption that each individual sample of read counts belongs to one of the two possible populations: Control/healthy or treatment/patient. Therefore, although each dataset is sampled from these populations, they are independent up to the limit for the algorithms of pseudorandom number generation (which is a standard simulation assumption). Since we are simulating a stochastic process, the Hellinger Divergence from each sample follows a different probability distribution, as indicated in Figure 1.

The reads of methylation counts are obtained as the product of coverage by the posterior methylation level. The R scripts for these simulations are available at the PSU GitLab: https://git.psu.edu/genomath/MethylIT_examples.

### 4.6. Experimental Methylation Datasets

The datasets of genome-wide methylated and unmethylated read counts (for each cytosine site) from normal CD19+ blood cell donors (NB) and from patients with pediatric acute lymphoblastic leukemia (PALL) were downloaded from the Gene Expression Omnibus (GEO) database [38]. DMPs were estimated for control (NB, GEO accession: GSM1978783 to GSM1978786) and for patients (ALL cells, GEO accession number GSM1978759 to GSM1978761) relative to a reference group of four independent normal CD19+ blood cell donors (GEO accession: GSM1978787 to GSM1978790). For the purposes of the analysis presented here, we only focused on the analysis of chromosome 9.

For the autism analysis [39], raw sequencing reads were downloaded from NCBI (GEO: GSE67615). The following methylome datasets from autistic children were retrieved from the GEO database:

***Group 1***: GSM1655495, GSM1655490, GSM1655488, GSM1652180, GSM1652179, GSM1652173, GSM1652172, GSM1652171, GSM1652157.

***Group 2***: GSM1655498, GSM1655497, GSM1655492, GSM1652167, GSM1652160, GSM1652156, GSM1652155, GSM1652154, GSM1652152, GSM1652149, GSM1652148.

Quality-controlled with *FastQC* (version 0.11.5), trimmed with TrimGalore! program(version 0.4.1) and Cutadapt (version 1.15), then aligned to the *Homo sapiens* reference genome (Homo_sapiens.GRCh37.dna.toplevel.fa) using Bismark (version 0.19.0) with bowtie2 (version 2.3.3.1). Bismark methylation extractor with default parameters was used to get methylation counts files (COV files).

## 5. Conclusions

The present study was designed to test the feasibility of performing clinical diagnostics of diseased individuals based on the application of signal detection theory and machine learning approaches to DNA methylation profiles obtained from patients. Simulation studies and analyses of reported methylome datasets from patients demonstrate the feasibility of such an approach. With the datasets tested, we were able to reach high classification performance that approaches the confidence level required for clinical diagnostics. We suggest that this system is appropriate for more extensive testing on a larger scale.

## Figures and Tables

**Table 1:** Summary of the datasets generated for simulation experiments.

Dataset	TV Average	Cytosine Sites/Sample	Samples/Group *	Cut-Point	Accuracy	Sen.	Spe.	FDR
1. Model building & cross-validation	0.0356	1,000,000	3	0.3500	1.0000	1.0000	1.0000	0.0000
2. Model building & cross-validation	0.1332	1,000,000	3	0.9404	0.9998	0.9997	1.0000	0.0000
3. Model building & cross-validation	0.1845	1,000,000	3	0.9251	1.0000	1.0000	1.0000	0.0000
4. External data for validation	0.0356	1,000,000	50	0.3500	1.0000	1.0000	1.0000	0.0000
5. External data for validation	0.1332	1,000,000	50	0.9404	0.9998	1.0000	1.0000	0.0000
6. External data for validation	0.1845	1,000,000	50	0.9251	1.0000	0.9999	1.0000	0.0000
7. Model building & cross-validation	0.0356	1,000,000	50	0.3500	1.0000	1.0000	1.0000	0.0000
8. Model building & cross-validation	0.1332	1,000,000	50	0.8667	1.0000	1.0000	1.0000	0.0000
9. Model building & cross-validation	0.1845	1,000,000	50	0.8306	1.0000	1.0000	1.0000	0.0000
10. External data for validation	0.0356	1,000,000	50	0.3500	1.0000	1.0000	1.0000	0.0000
11. External data for validation	0.1332	1,000,000	50	0.8667	1.0000	1.0000	1.0000	0.0000
12. External data for validation	0.1845	1,000,000	50	0.8306	1.0000	1.0000	1.0000	0.0000

* Four independent control samples were used to build the reference sample (the centroid) for each simulation 1 to 6; while twenty were used for simulations 7 to 12. All the R scripts for these simulations are available at https://git.psu.edu/genomath/MethylIT_examples.

**Table 2:** Classification performance of four different classifier models based on DMPs identified from the placenta tissue of children with autism by signal detection.

Group	Accuracy	Sensitivity	Specificity	Detection Rate	FDR	Classifier
G1	0.995	0.998	0.966	0.906	0.004	PCA-QDA
G2	1.000	1.000	1.000	0.994	0.000	PCA-QDA
G2 pred. G1	0.935	0.996	0.332	0.904	0.064	LDA
G1 pred. G2	1.000	1.000	1.000	0.994	0.000	LDA

G1: The classifier models built based on Group 1 individuals (60% of DMP were used as training set), and then used to classify the rest (40%) within Group1. G2: The classifier models built based on Group 2 individuals (60% of DMP were used as training set), and then used to classify the rest (40%) within Group 2. G2 pred. G1: The classifier models built based on Group 2 individuals were used to classify the individuals within Group 1. G1 pred. G2: The classifier models built based on Group 1 individuals were used to classify the individuals within Group 2. All four classifier models performed well.

## Supplementary Materials

Supplementary materials can be found at https://www.mdpi.com/1422-0067/20/21/5343/s1.

## Abbreviations

ADP	Autistic developing placenta
CDM	Cytosine DNA methylation
DMPs	Differentially methylated position
FDR	False discovery rate
FT	Fisher’s exact test
HD	Hellinger divergence
ML	Machine learning
PALL	Pediatric acute lymphoblastic leukemia
RMST	Root mean squared test
SD	Signal detection (used to denote the signal detection approach)
TDP	Typically developing placenta
TV	Total variation distance (absolute value of methylation level difference)
