# Supplementary material for: Discrimination of DNA Methylation Signal from Background Variation for Clinical Diagnostics
Source: Int J Mol Sci. 2019 Oct 27;20(21):5343. doi: 10.3390/ijms20215343 (PMC6862328; doi:10.3390/ijms20215343)
Supplement: Supplementary file 1 [file ijms-20-05343-s001.pdf]

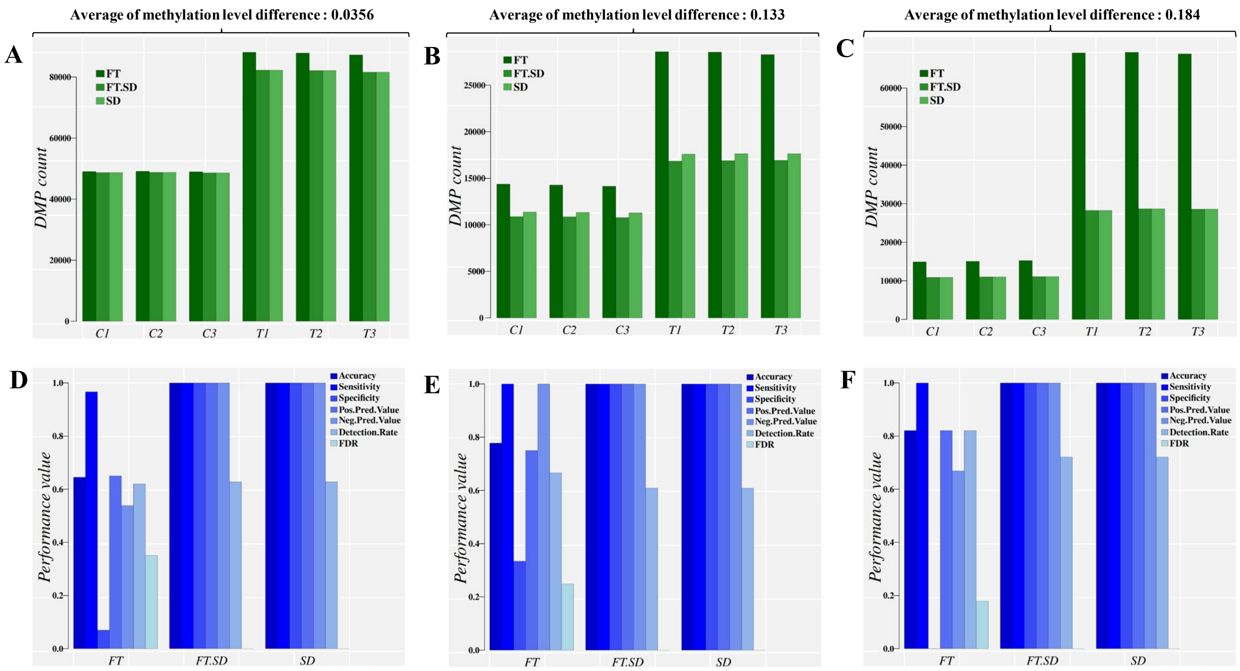

Figure S1. Classification performance for control and treatment DMPs obtained at three different averages of absolute methylation level differences following Fisher's exact test output and provided with information from the signal detection approach (FT.SD). Panel (**A**) to (**C**), bar-plots of DMP counts. Panels (**D**) to (**F**), classification performance evaluation.

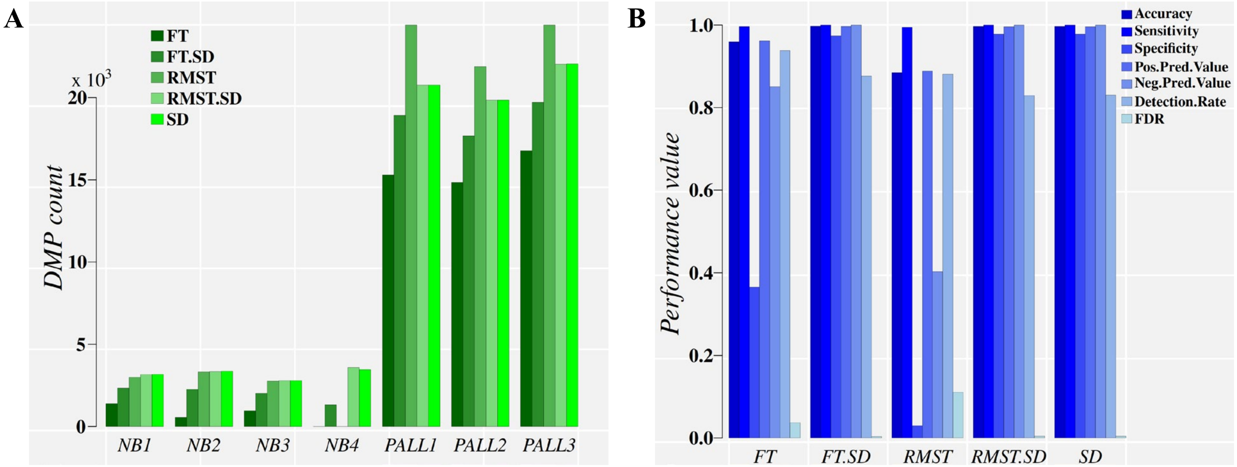

Figure S2. Classification performance for control and treatment DMPs obtained on Chromosome 9 from PALL patients after providing signal detection (SD) information to FT and RMST approaches (FT.SD and RMST.SD). Panel (**A**), bar-plots of DMP counts. Panel (**B**), classification performance evaluation.

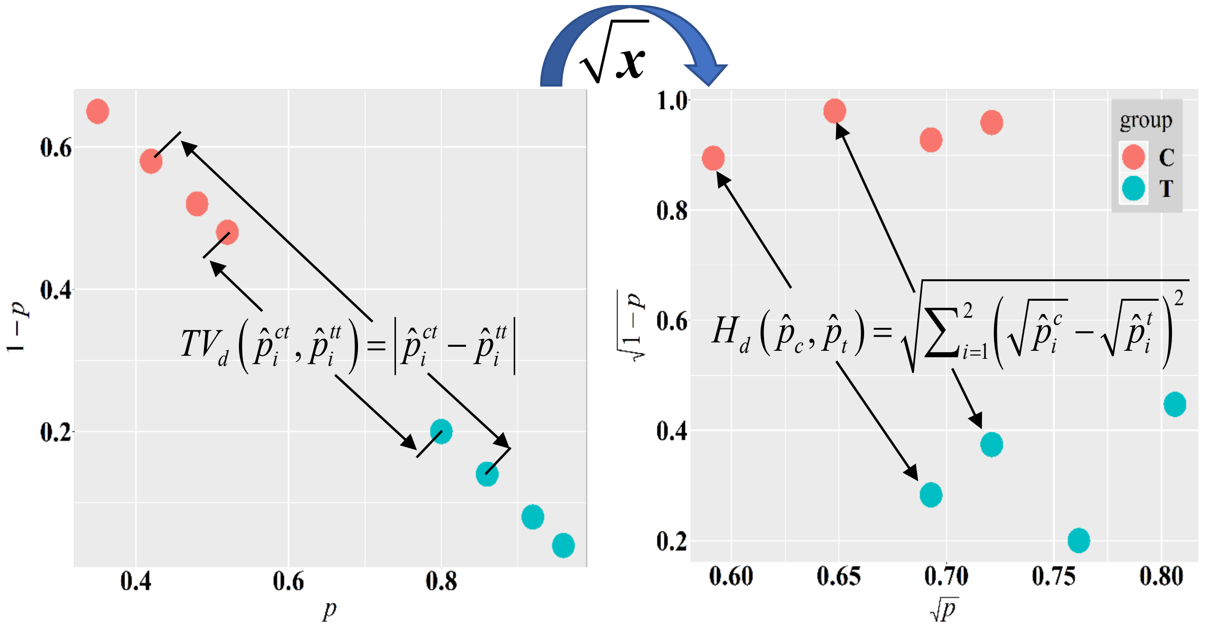

Figure S3. The measurement of methylation divergence. Mapping of the space $(p, 1-p)$ into the space $(\sqrt{p}, \sqrt{1-p})$ unveils the relationship between absolute value of methylation level difference (total variation distance, *TV*) and Hellinger divergence. In the space $(p, 1-p)$, TV derives from the Manhattan distance. After the mapping $(p, 1-p) \rightarrow (\sqrt{p}, \sqrt{1-p})$, frequently used in statistical analyses of biological datasets, the Hellinger distance in the space $(p, 1-p)$ derives from the Euclidean distance in the space $(\sqrt{p}, \sqrt{1-p})$.

Table S1. Estimation of the false positive rate empirical upper bound for four different approaches with three simulated datasets, with methylation level difference = 0.0356, 0.133 and 0.184, respectively.

	$TV_d^{Cut} = 0$ TV_d^{OptCut} **by ECDF**	$TV_d^{Cut} = 0$ TV_d^{OptCut} **by nonliner fit**	TV_d^{Cut} **by ECDF** TV_d^{OptCut} **by nonliner fit**	TV_d^{Cut} **by ECDF** TV_d^{OptCup} **by Youden index**
**Mean of absolute difference of methylation levels: 0.0356**				
**Min.**	0.037743	0.053417	0.016238	0.000000
**1st Qu.**	0.038032	0.053718	0.016399	0.000000
**Median**	0.038721	0.059094	0.021225	0.000000
**Mean**	0.038782	0.059114	0.021290	0.000000
**3rd Qu.**	0.039529	0.064474	0.026160	0.000000
**Max.**	0.039878	0.064894	0.026464	0.000000
**Mean of absolute difference of methylation levels: 0.133**				
**Min.**	0.002237	0.001629	0.000000	0.003887
**1st Qu.**	0.002324	0.001768	0.000000	0.004066
**Median**	0.003055	0.002210	0.000000	0.005784
**Mean**	0.003060	0.002218	0.000000	0.005805
**3rd Qu.**	0.003791	0.002723	0.000000	0.007559
**Max.**	0.003967	0.002864	0.000000	0.007720
**Mean of absolute difference of methylation levels: 0.184**				
**Min.**	0.000084	0.000013	0.000000	0.002499
**1st Qu.**	0.000104	0.000020	0.000000	0.002597
**Median**	0.000167	0.000027	0.000000	0.005803
**Mean**	0.000172	0.000029	0.000000	0.005887
**3rd Qu.**	0.000238	0.000036	0.000000	0.009166
**Max.**	0.000275	0.000051	0.000000	0.009345

Any cytosine site $\boldsymbol{k}$ for which $H^{OptCut} \le H_k^j$ and $TV_k^j < TV_d^{OptCut}$ (*j* = *CT*, *TT*) was considered a false positive DMP. Four different DMP identification approaches within MethylIT were tested: 1. Optimal TV_d cut (TV_d^{OptCut}) estimation based on the empirical cumulative distribution function (***ECDF***) with no minimal TV_d cutoff ($TV_d^{Cut} = 0$); 2. TV_d^{OptCut} estimation based on the nonlinear fit of TV_d probability distribution, and with no minimal TV_d cut-off ($TV_d^{Cut} = 0$); 3. TV_d^{OptCut} estimation based on the nonlinear fit of TV_d probability distribution, with minimal TV_d cutoff estimated by ***ECDF*** (Methyl-IT default); and 4. TV_d^{OptCut} estimation based on Youden index, with minimal TV_d cutoff estimated by ***ECDF***. The R scripts for these simulations are available at https://git.psu.edu/genomath/MethylIT_examples.

We assume that the expected FPR for a signal detection approach has the following upper bounds:

$$FPR \le 1 - P\left(TV_d \le TV_d^{OptCup}\right) \text{ (S1) and } FPR \le 1 - P\left(H \le H^{OptCut}\right) \text{ (S1),}$$

where $P\left(TV_d \le TV_d^{OptCup}\right)$ denotes the probability to observe a total variation distance value $TV_d \le TV_d^{OptCup}$, TV_d^{OptCup} stands for some optimal cut-point value $TV_d^{OptCup} \ge max\left(TV_{\alpha=0.05}^{CT}, 0.25\right)$, and $max\left(TV_{\alpha=0.05}^{CT}\right)$ denotes the maximum for critical values $TV_{\alpha=0.05}^{CT}$ of variable TV_d at $\alpha = 0.05$ found in the control group (*CT*). $P\left(H \le H^{OptCut}\right)$ stands for the probability to observe a Hellinger divergence $H \le H^{OptCut}$ for some optimal cut-point value $min\left(H_{\alpha=0.05}^{TT}\right) \le H^{OptCut}$, where $min\left(H_{\alpha=0.05}^{TT}\right)$ denotes the minimum of the critical values $H_{\alpha=0.05}^{TT}$ of variable H for $\alpha = 0.05$ found in the treatment group (*TT*).

According to relevant statistical models for the probability distribution of information divergences for methylation levels, the probability to observe a methylation event $TV_d > max\left(TV_{\alpha=0.05}^{CT}, 0.25\right)$ induced by random noise is lower than 0.05.

Methyl-IT function *getPotentialDIMP* is applied to restrict analysis to the subset of cytosine sites from control and treatment that satisfy the inequalities: $TV_d^{Cut} \le TV_d^{CT}$, $TV_d^{Cut} \le TV_d^{TT}$, $H_{\alpha=0.05}^{CT_j} < H_k^{CT}$ and $H_{\alpha=0.05}^{TT_j} < H_k^{TT}$ (S3), where TV_d^{Cut} is a numerical TV_d cut-value specified by the user to filter cytosine sites; $H_{\alpha=0.05}^{CT_j}$ and $H_{\alpha=0.05}^{TT_j}$ represent critical values at $\alpha = 0.05$ for the Hellinger divergence values for each individual j from control and treatment, respectively. Cytosine sites that hold the inequalities (S3) are named potential DMPs (***pDMPs***).

The signal detection step (optimal cut-point estimation) is carried out on sets of ***pDMPs***. As a result, in the context of Methyl-IT pipeline, application of the inequalities (S3) implies the assumption:

$$FPR \le 1 - P\left(TV_d \le TV_d^{OptCup} \cap H \le H^{OptCut}\right) \quad \text{(S4)}$$

That is, DMPs are searched in the space of events $TV_d > TV_d^{OptCup} \cap H > H^{OptCut}$. Equation S4 sets an upper bound for the expected value of FPR in the signal detection approach based on information from TV_d and H probability distributions. Hence, we can write: $FPR \le FPR_H$ and

$$FPR_H = 1 - P\left(TV_d \leq TV_d^{OptCup} \cap H \leq H^{OptCut}\right) \text{ (S5).}$$

Thus, we can only estimate upper bounds for ***FPR***, since we do not have theoretical or experimental evidence to support specific optimal TV_d^{OptCup} and H^{OptCut} values valid for each individual from control and treatment groups.

To evaluate the magnitude of ***FPR*** we can follow the following steps:

i) To estimate cutpoint H^{OptCut} and TV_d^{OptCut} in a training dataset. H^{OptCut} will be the Youden index, estimated for the classification of DMPs into two classes, control and treatment. All the information used in the estimation of H^{OptCut} comes from the probability distribution of ***H***. Likewise, all the information used in the estimation of TV_d^{OptCut} comes from the probability distribution of TV_d. The optimal cut-point estimation using ML provided in Methyl-IT cannot be used here.

ii) To apply the cutpoints H^{OptCut} and TV_d^{OptCut} on independent external datasets of control and treatment groups (same external datasets from Table 1). Any cytosine site ***k*** for which $H^{OptCut} \leq H_k^j$ and $TV_k^j < TV_d^{OptCut}$ (*j* = *CT*, *TT*) will be considered a ***false positive*** (***FP***) DMP. That is, TV_d^{OptCut} provides the critical threshold to identify false DMPs.

iii) To estimate $FPR_H = \dfrac{FP}{N}$, where ***N*** is the number of negatives; in our case, all the cytosine sites ***k*** (from external datasets) for which $TV_k^j < TV_d^{OptCut}$.

Four different approaches were followed for the application of the above heuristic:

a) Estimation and application of $TV_d^{OptCup} = \boldsymbol{max}\left(TV_{\alpha=0.05}^{CT}\right)$ based on the empirical cumulative distribution function (*ECDF*). $TV_d^{Cut} = 0$ (Methyl-IT function getPotentialDIMP).

b) Estimation and application of $TV_d^{OptCup} = \boldsymbol{max}\left(TV_{\alpha=0.05}^{CT}\right)$ based on the best nonlinear fit of TV_d using Methyl-IT function: nonlinearFitDist. $TV_d^{Cut} = 0$.

c) Default Methyl-IT pipeline: $\boldsymbol{TV_d^{Cut}} = \boldsymbol{max}\left(\boldsymbol{ECDF}: \boldsymbol{TV_{\alpha=0.05}^{CT}}\right)$ (argument of *getPotentialDIMP*) was estimated based on the *ECDF*. $\boldsymbol{TV_d^{OptCup}} = \boldsymbol{max}\left(\boldsymbol{TV_{\alpha=0.05}^{CT}}\right)$ was estimated based on the best nonlinear fit of $\boldsymbol{TV_d}$ using Methyl-IT function *nonlinearFitDist*.

d) $\boldsymbol{TV_d^{Cut}} = \boldsymbol{max}\left(\boldsymbol{ECDF}: \boldsymbol{TV_{\alpha=0.05}^{CT}}\right)$ (argument of *getPotentialDIMP*) was estimated based on the *ECDF*. $\boldsymbol{TV_d^{OptCup}}$ corresponds to the Youden index, the cut-point for classification of DMPs into two classes, control and treatment DMPs, based on distribution of $\boldsymbol{TV_d}$.

Estimation of the ***FPR*** empirical upper bounds on simulated datasets are presented in Table S1. While the FDR rate increases in response to narrowing of overall methylation level difference (from 0.184 to 0.0356), all of the estimation remains below 0.05. This result suggests that we gain a greater biological confidence for ***FPR*** below 0.05 by including information from the $\boldsymbol{TV_d}$ and $\boldsymbol{H}$ probability distributions.
